# Solitary Sacral Osteochondroma Causing Postural Difficulty in a Young Female: A Case Report and a Review of the Literature

**DOI:** 10.7759/cureus.6470

**Published:** 2019-12-26

**Authors:** Sujit K Tripathy, Saurav N Nanda, Mukund K Sable, Sunil Doki, Sandeep Velagada

**Affiliations:** 1 Orthopaedics, All India Institute of Medical Sciences, Bhubaneswar, IND; 2 Orthopaedics, Kalinga Institute of Medical Sciences, Bhubaneswar, IND; 3 Pathology, All India Institute of Medical Sciences, Bhubaneswar, IND

**Keywords:** spine, benign tumor, en-bloc excision, osteochondroma, sacral tumor

## Abstract

Sacral osteochondromas are rare tumors, and a handful of cases have been reported in the literature. The clinical manifestations of sacral osteochondral may vary from a painless mass to a complete neurological deficit. We report a case of sacral osteochondroma arising from S2-3 lamina causing difficulty in lying down in the supine position and sitting. Computed tomographic (CT) scan and magnetic resonance imaging (MRI) delineated the extent of the lesion and confirmed it to be a benign tumor. It was excised en bloc through a posterior midline approach. At two years follow-up, the patient was asymptomatic and the radiograph did not show any evidence of recurrence. To the best of our knowledge, this is the second case report on sacral osteochondroma, which caused postural difficulty in a young female.

## Introduction

Bony growth in the sacrum can be either benign or malignant or may be a metastatic lesion. Because of the inaccessibility and proximity to the major neurovascular structures, surgical excision is often difficult. Approximately 10% of the benign tumors or pseudotumors are seen in the sacrum, and the common benign tumors are giant cell tumors (60%), aneurysmal bone cysts (4%), and osteoblastomas [1]. Osteochondroma, the most common benign tumor of the long bones, is rarely seen in the sacrum [2-4]. Few cases have been reported in the literature, with variable manifestations [5-6]. It may compress the spinal nerve roots and produce neurological symptoms or may remain as an asymptomatic painless mass [2,7-9]. However excessive growth of the mass on the posterior aspect may cause difficulty in sleeping and sitting [10]. We report a case of solitary sacral osteochondroma in a young female who could not lie down on her back due to the pressure of the mass and sought treatment to get relief from the mechanical pressure symptom.

## Case presentation

**Figure 1:**
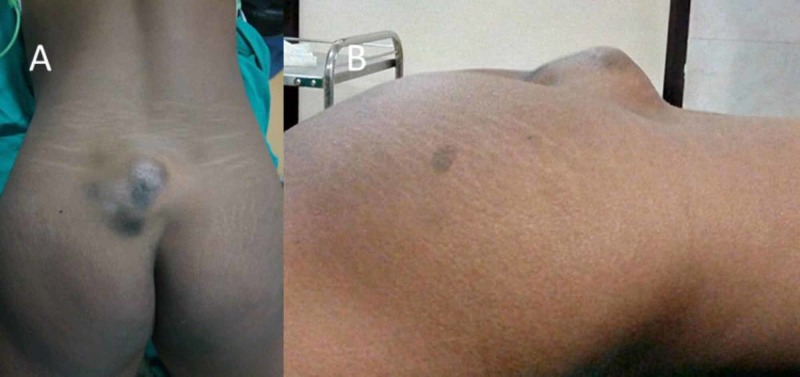
Clinical photograph of the back of a young female showing a bony outgrowth from the sacrum

A 19-year-old young female presented with a painless, palpable bony swelling over her lower back region (Figure 1). It was progressively increasing in size for the last 4 years and had grown to such an extent that she was uncomfortable in lying down on her back for the last 4 months. On examination, a hard non-tender globular mass of size 8 x 5 x 5 cm was palpable in the midline of the S2-3 region, more towards left, with a smooth margin, and the overlying skin was non-adherent. The overlying skin was thick and showed black discoloration. There was no neural deficit, and the bowel/bladder functions were normal.

**Figure 2:**
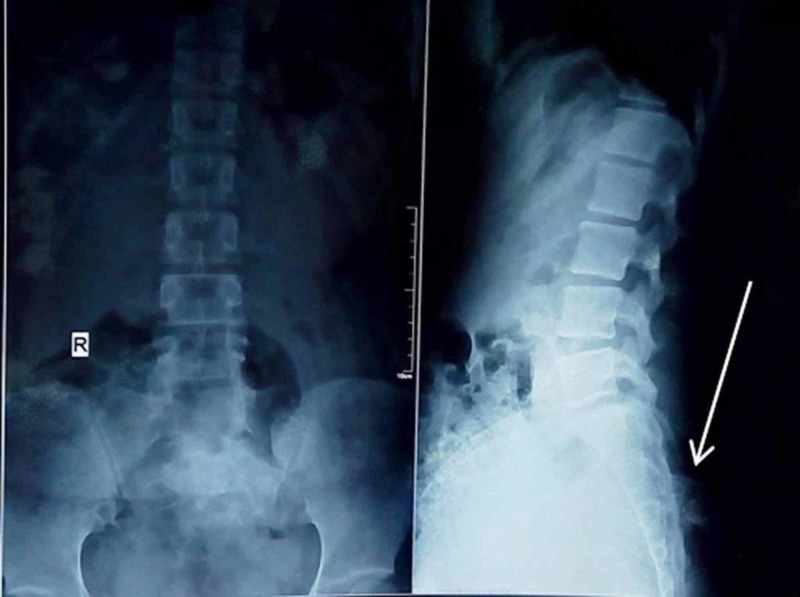
Radiograph showing a bony growth arising from the S2-3 level

**Figure 3:**
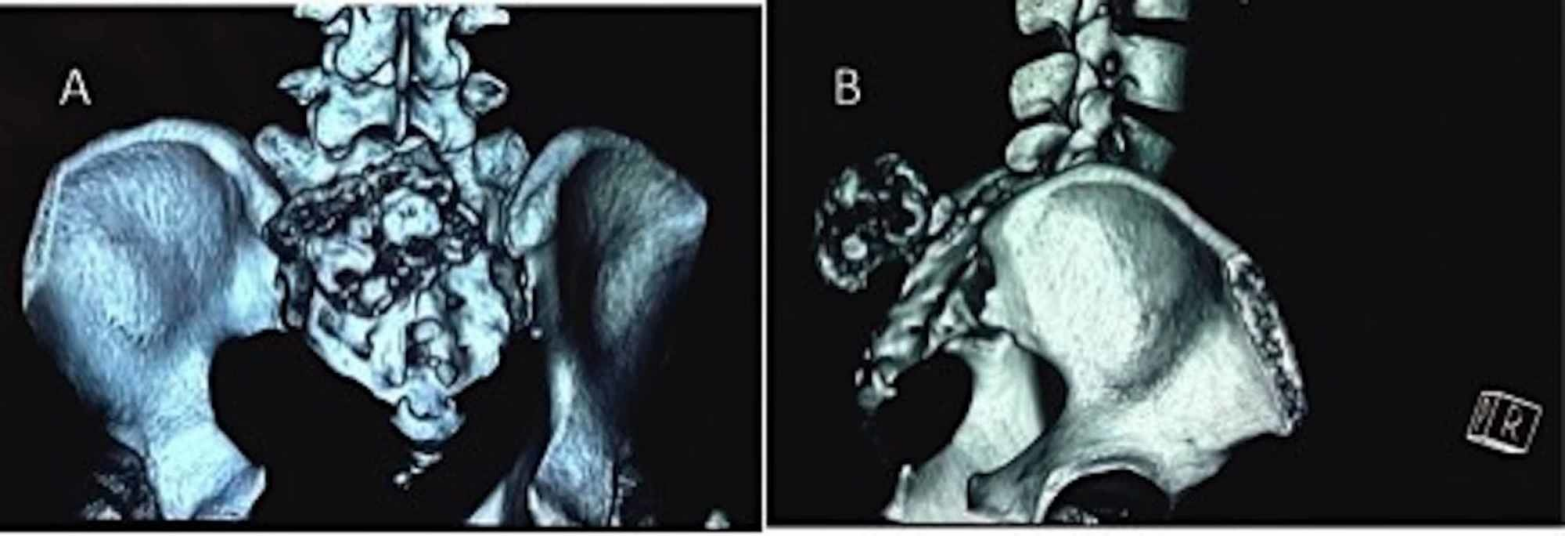
CT scan clearly delineates the growth on the left side of the sacrum arising from the S2-3 level

**Figure 4:**
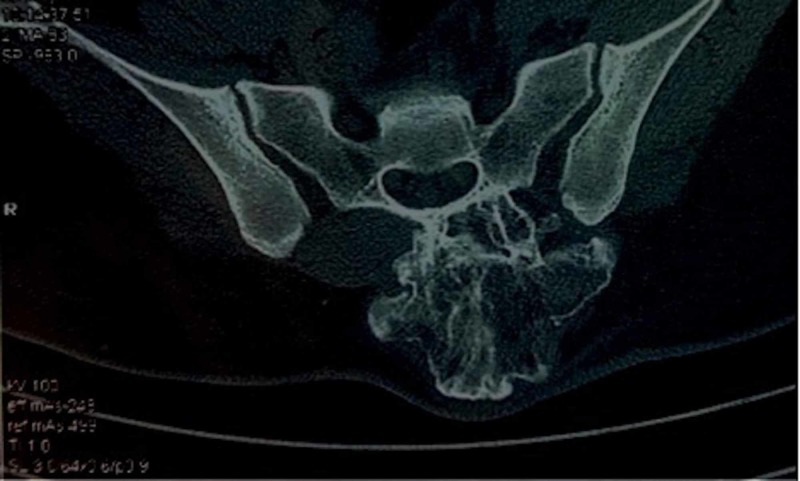
Transverse cut section of the CT scan shows a cauliflower-like bony outgrowth arising from the posterior aspect of the sacrum on the left side

**Figure 5:**
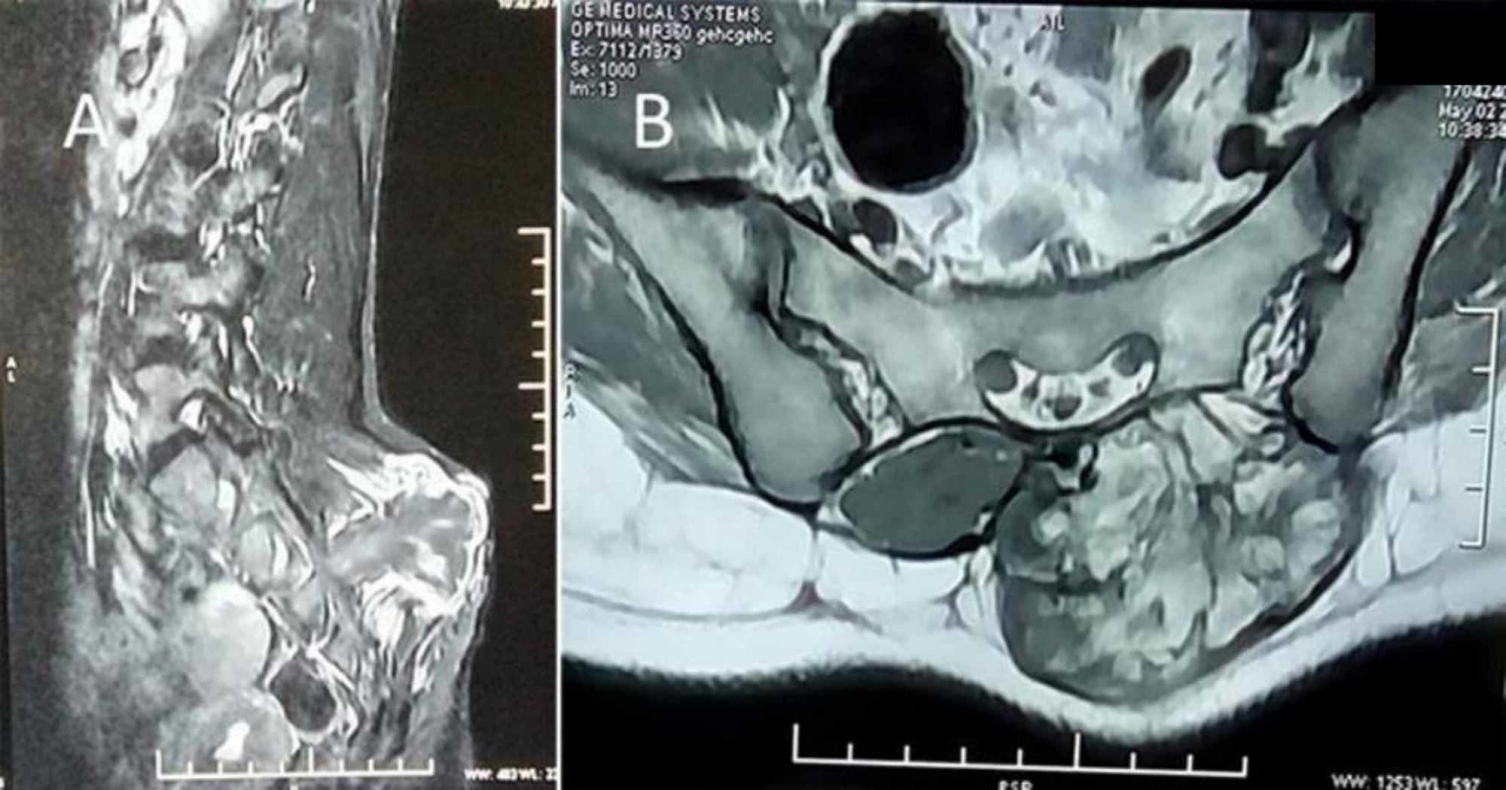
MRI shows a growth with a hypointense rim and a hyperintense center on T1- and T2-weighted images, suggestive of osteochondroma

Radiograph of the lumbosacral spine showed a bony outgrowth in the posterior aspect of the S2-3 region (Figure 2). On skeletal survey, no similar lesion was found in any other part. Computed tomographic (CT) scan was performed to delineate bony details (Figures 3 and 4). MRI showed a bony outgrowth of size 81 x 51 x 54 mm arising from posterior elements of the S2-3 region in the midline (more towards left side). It was well circumscribed by soft tissue envelope. The lesion showed a hypointense rim with a heterogeneously hyperintense center on T1- and T2-weighted images, indicating marrow suggestive of osteochondroma (Figure 5). After a detailed discussion with the patient and her relatives, excisional biopsy was planned for local tumor control as well as for diagnostic purpose.

**Figure 6:**
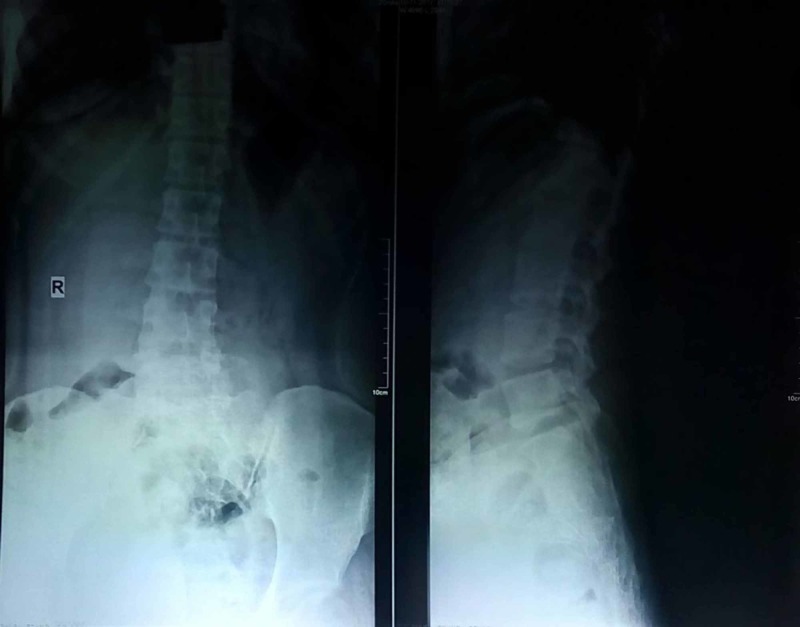
Three-year follow-up radiograph shows no evidence of recurrence or reappearance of the sacral lesion

The tumor was approached through a posterior midline incision. The whole tumor was exposed, and en bloc excision was performed from the base of the tumor along with the cartilaginous cap from the conjoint lamina of the S2-3 vertebra keeping a healthy margin (Figure 6). Care was taken in the whole procedure to prevent damage to the neurovascular structures. The wound was closed in layers with a negative suction drain. Postoperative radiograph showed complete excision of the bony mass, and the patient got relief from the pressure symptoms.

**Figure 7:**
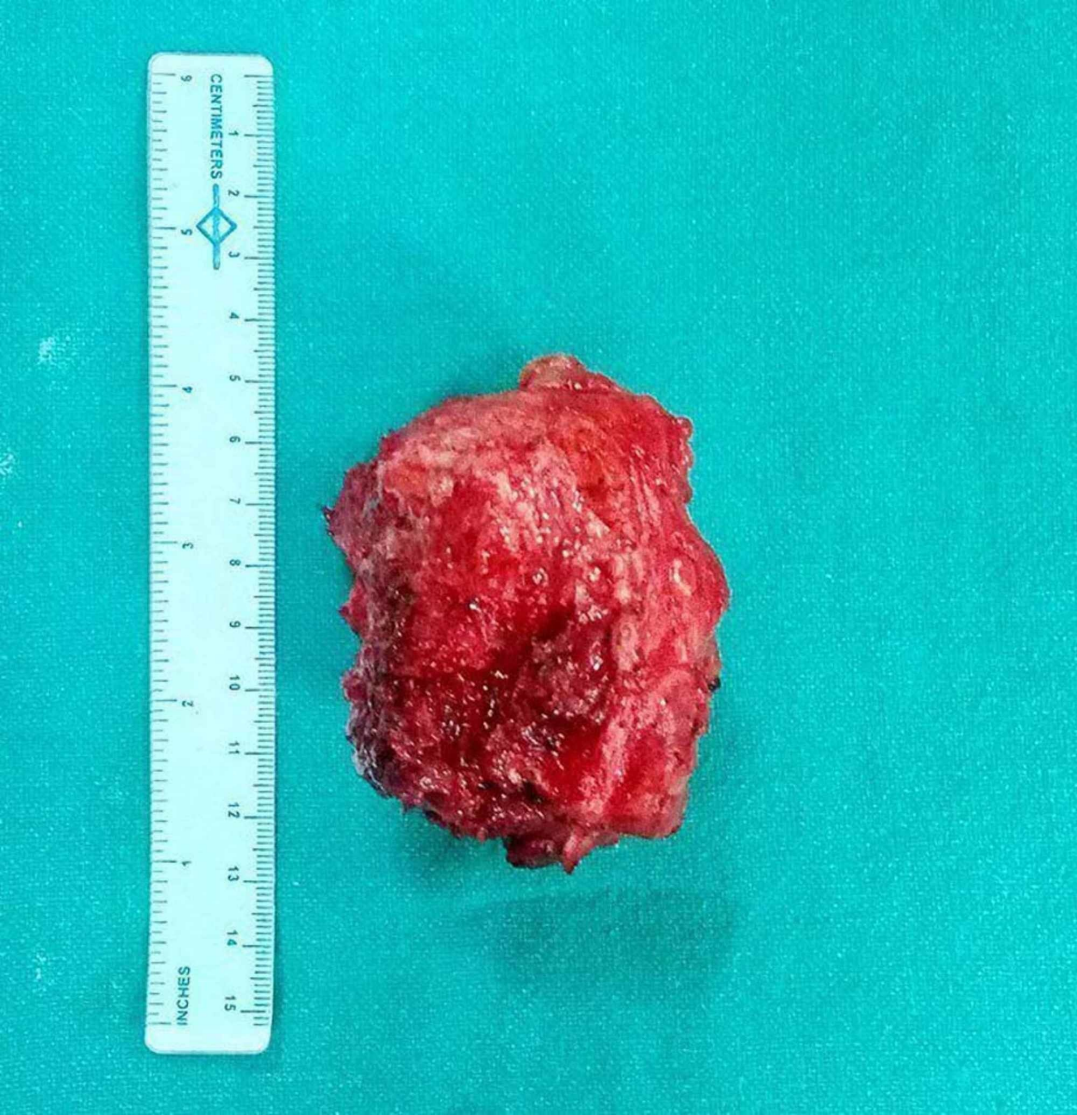
Excised specimen of osteochondroma

**Figure 8:**
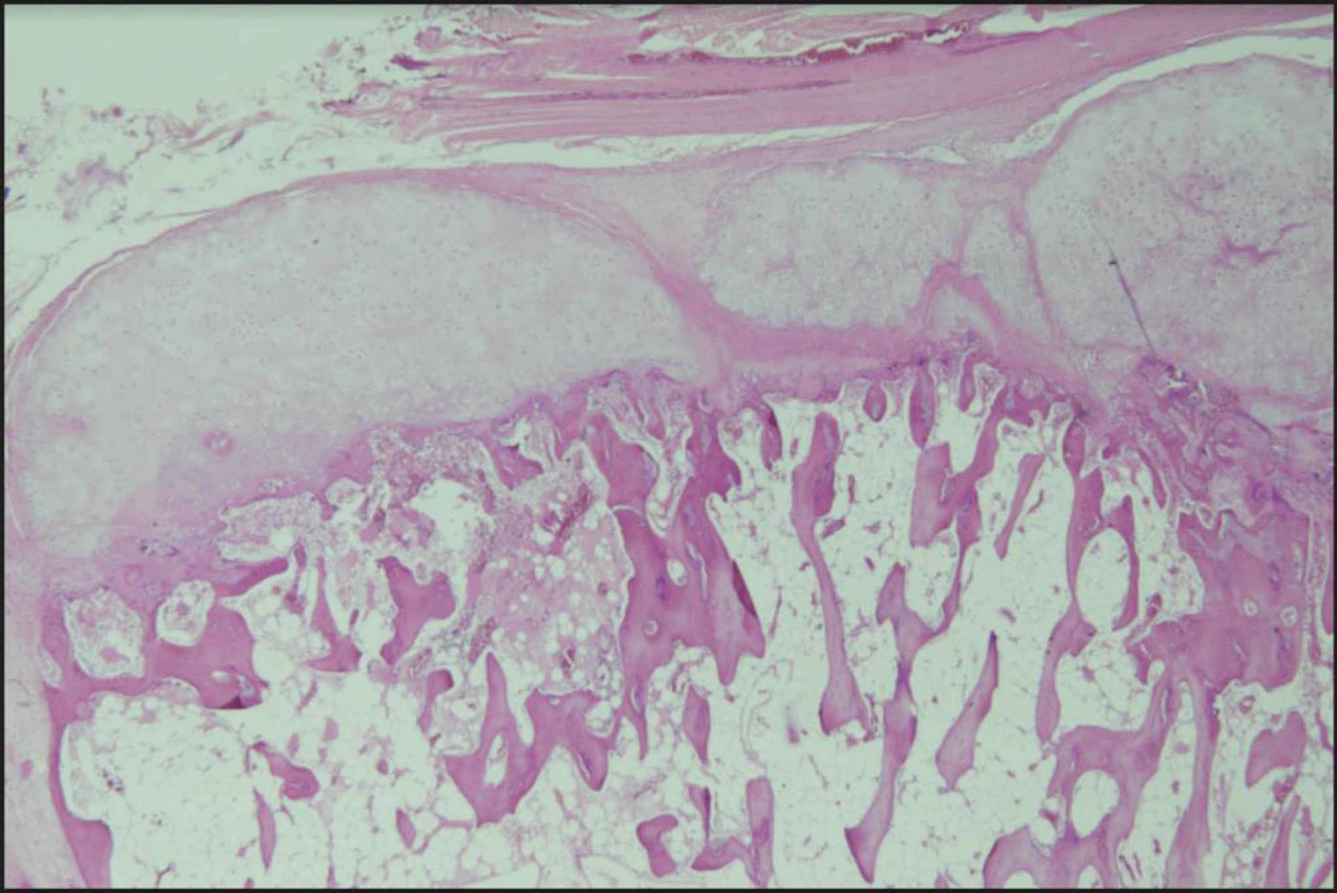
Histopathology showing the cartilaginous cap with the underlying mature bone trabeculae and evidence of endochondral ossification suggestive of osteochondroma

Histopathology report showed a benign tumor with mature bone trabeculae and endochondral ossification with a cap of hyaline cartilage, confirming the diagnosis of osteochondroma (Figure 7). On follow-up at 6 weeks, 3 months, 6 months, 12 months, and 24 months, there was no clinical and radiological recurrence (Figure 8) of the disease, and no neurological deficit was detected.

## Discussion

Osteochondroma is an abnormal development of growth plate that separates and grows progressively into a bony protuberance by endochondral ossification. It is covered by the cartilaginous cap, and the marrow cavity is in continuation with the parent bone [1]. It is the most common benign tumor of the bone and is usually seen in the metaphysis of long bones. However, osteochondroma in the spine is uncommon. In all age groups, spinal osteochondroma has been reported in only 3% of cases [2,11]. The predominant sites for osteochondroma in the spine are the cervical and upper thoracic vertebra [2,11]. Sacral osteochondroma is very rare, accounting for only 0.5% cases [2,11].

After a search of major databases such as PubMed/Medline, EMBASE, SCOPUS, Google scholar, and Biomed central for sacral osteochondroma, we could retrieve nine articles that reported 14 cases till date. The details of the cases and their management including the present case (15th case) were tabulated to gather information on sacral osteochondroma (Table 1). It was observed that the S1 vertebra is commonly involved in sacral osteochondroma. Symptoms of sacral osteochondroma depend on its location and extent [2,10]. The symptoms may vary from a painless mass to a substantial neurological deficit. All cases except that of Baruah et al. presented with a neurological deficit or radiculopathy due to spinal nerve compression [2,4,7-10]. The patient in our study did not have any neurological involvement and produced only pressure symptoms on the skin that caused difficulty in lying down. After review of the literature, it seems that neurological involvement in sacral osteochondroma is common in the upper sacral region, with a predominant anterior growth that protrudes into the spinal canal or impinges on the nerve root [2,4,7-9]. Lower sacral osteochondroma with posterior outgrowth usually has a mass effect, causing discomfort upon lying down and sitting [10]. Patients with sacral tumors from posterior elements without neurological compromise may present late as no serious consequence such as neurological deficit is seen. Our patient had neglected the growth for four years and presented very late with a huge mass.

**Table 1:** Summary of published literature on sacral osteochondroma F, female; M, male

References	No. of cases	Age/ sex	Level	Location	Size	Presentation	Treatment
Pugh et al. [5]	1	-	-	-	-	-	-
Sung et al. [6]	2	-	Below S3	-	-	-	Excision of tumor through a posterior approach
Hanakita and Suzuki [7]	1	42 years/F	L5-S1	Lamina of S1	-	Low back ache and cauda equina compression	Hemilaminectomy of left L4-L5 and right L5-S1
Agrawal et al. [8]	1	14 years/M	S1	Right sacral ala	5x5 cm	Low back ache with right lower limb radiculopathy, neurological deficit	Excision of tumor through a posterior approach
Samartzis and Marco [2]	1	11 years/M	S2	Anterior surface of the S2 lamina	2.5 cm	Right posterior thigh pain	En bloc excision with right S1-S4 laminectomy
Chin and Kim [9]	1	54 years/F	Left anterior surface of the sacral ala	Left sacral ala	-	Low back ache with left lower limb radiculopathy, neurological deficit	En bloc excision through a retroperitoneal abdominal approach
Kuraishi et al. [4]	1	63 years/F	S1	Superior articular Process of S1	-	Foot drop, numbness	Right partial hemilaminectomy at the L5-S1 level
Sciubba et al [3]	5	-	S1 (4 patients) L5-S3 (1 patient)	-	4 cm^3^,120 cm^3^, 120 cm^3^, 27 cm^3^ 616 cm^3^	-	En bloc excision, one recurrence
Baruah et al. [10]	1	21 years/M	Conjoint lamina of S3-S4	Left of the midline	36x22 mm	Painless mass, uncomfortable in lying down (mechanical symptom)	Excision of tumor through a posterior approach
Present case	1	19 years/F	Conjoint lamina of S2-S3	Left of the midline	81x51x54 mm	Painless mass, difficulty in lying down	Excision of tumor through a posterior approach

Although X-ray is the primary modality of imaging, it may not truly reveal the extent of the lesion in sacral tumors. CT scan clearly delineates the lesion. MRI must be performed to look for cartilaginous cap thickness of the lesion and for malignant transformation. In case of neurological involvement, MRI will reveal the nerve impingement or canal compromise [1,11].

Surgical treatment of sacral osteochondroma is challenging due to its proximity to the sacral plexus, major blood vessels, pelvic organs, and sacroiliac joint. The excision of osteochondroma protruding into the spinal canal or sacral hiatus needs meticulous dissection as these nerve roots are mainly responsible for genitourinary and bowel/bladder function [2]. En bloc excision usually results in complete excision of the tumor, and recurrence is very uncommon [1,3]. For posterior lesion in the midline or the paramedian region, posterior midline incision is adequate. However, extreme lateral lesion on the back can be excised with further extension into the iliac crest in a curvilinear fashion. Complete excision of tumor mass along with the cartilage cap and periosteum decreases the chances of recurrence rate [3]. Sciubba et al. have mentioned only one sacral osteochondroma recurrence in the literature till date [3]. The tumor in their case was small (4 cm3) and was a grade 1 benign tumor. Despite an en bloc excision, the tumor showed recurrence. The contention of Morton may hold true for the reappearance of osteochondroma. Morton believed that the reappearance of osteochondroma is not a recurrence of the disease always, rather it is the appearance of another tumor in juxtaposition to the initial one [12]. This concept is in agreement with the multiplicative growth nature of hereditary multiple exostoses (HMEs). New research has clarified that both solitary osteochondroma and HMEs are the same spectrum of the disease from the same family of tumor suppressor gene encoding EXT [3]. Thus, even with solitary lesion, a new tumor may arise close to the initial lesion even after its excision and without a full onset of HME. Our patient has not shown any recurrence or reappearance of the tumor even after two-year follow-up, but the tumor reappearance in future cannot be assured as it needs a long follow-up.

## Conclusions

Solitary sacral osteochondromas with posterior growth may remain asymptomatic for a long time. Such tumors may attain a large size producing a mass effect on the overlying skin, thus making it difficult for the patient to lie down in the supine position. En bloc excision of the tumor provides immediate relief and less chances of recurrence.
